# A novel mouse model of tuberous sclerosis complex (TSC): eye-specific *Tsc1*-ablation disrupts visual-pathway development

**DOI:** 10.1242/dmm.021972

**Published:** 2015-12-01

**Authors:** Iwan Jones, Anna-Carin Hägglund, Gunilla Törnqvist, Christoffer Nord, Ulf Ahlgren, Leif Carlsson

**Affiliations:** Umeå Center for Molecular Medicine (UCMM), Umeå University, Umeå 901 87, Sweden

**Keywords:** TSC, Retina, Hamartoma

## Abstract

Tuberous sclerosis complex (TSC) is an autosomal dominant syndrome that is best characterised by neurodevelopmental deficits and the presence of benign tumours (called hamartomas) in affected organs. This multi-organ disorder results from inactivating point mutations in either the *TSC1* or the *TSC2* genes and consequent activation of the canonical mammalian target of rapamycin complex 1 signalling (mTORC1) pathway. Because lesions to the eye are central to TSC diagnosis, we report here the generation and characterisation of the first eye-specific TSC mouse model. We demonstrate that conditional ablation of *Tsc1* in eye-committed progenitor cells leads to the accelerated differentiation and subsequent ectopic radial migration of retinal ganglion cells. This results in an increase in retinal ganglion cell apoptosis and consequent regionalised axonal loss within the optic nerve and topographical changes to the contra- and ipsilateral input within the dorsal lateral geniculate nucleus. Eyes from adult mice exhibit aberrant retinal architecture and display all the classic neuropathological hallmarks of TSC, including an increase in organ and cell size, ring heterotopias, hamartomas with retinal detachment, and lamination defects. Our results provide the first major insight into the molecular etiology of TSC within the developing eye and demonstrate a pivotal role for *Tsc1* in regulating various aspects of visual-pathway development. Our novel mouse model therefore provides a valuable resource for future studies concerning the molecular mechanisms underlying TSC and also as a platform to evaluate new therapeutic approaches for the treatment of this multi-organ disorder.

## Abstract (precis)

**Editors' choice:** Conditional deletion of *Tsc1* in the eye results in hamartoma formation and defects in retinal ganglion cell development – a novel mouse model providing insights into visual pathway involvement in TSC.

## INTRODUCTION

Genetic disruption during the formation of the central nervous system (CNS) is one of the underlying causes of neurodevelopmental deficits; the incidence rate of such disruptions is high within multi-organ syndromes such as tuberous sclerosis complex (TSC) (OMIM191100) (Han and Sahin, 2011; Smalley, 1998; Thiele, 2004). TSC is an autosomal dominant disorder that is caused by inactivating point mutations in either the *TSC1* (9q34) or the *TSC2* (16p13.3) genes. The protein products of *TSC1* and *TSC2* (hamartin and tuberin, respectively) form a heterodimeric complex that is stabilised by a third protein partner (TBC17D). This complex negatively regulates cell growth and proliferation through a canonical signalling pathway involving Ras homologue enriched in brain (Rheb) and the mammalian target of rapamycin complex 1 (mTORC1). TSC is best characterised by the presence of benign tumours (called hamartomas) in affected organs due to uncontrolled cell growth driven by mTORC1 hyperactivity. Hamartomas commonly present as cardiac rhabdomyomas, renal angiomyolipomas and facial angiofibroma. At the neuropathological level, hamartomas take the form of white matter radial migration lines (RMLs), subependymal nodules (SENs), subependymal giant cell astrocytes (SEGAs) and cortical tubers (Capo-Chichi et al., 2013; Cheadle et al., 2000; Dibble et al., 2012; DiMario, 2004; Garami et al., 2003; Han and Sahin, 2011; Jones et al., 1999; Kwiatkowski and Manning, 2005; Samueli et al., 2015). Individuals with TSC also present with a myriad of complex neurological deficits, with autism and epilepsy being prevalent amongst affected individuals. These observations clearly demonstrate that TSC is a multifaceted syndrome in which multiple CNS regions contribute to both the neurological and behavioural components (Costa-Mattioli and Monteggia, 2013; Han and Sahin, 2011; Jeste et al., 2008; Smalley, 1998).

The generation of rodent models has proved to be a robust approach for establishing the molecular etiology underlying TSC. Germline deletion of either *Tsc1* or *Tsc2* is embryonic lethal owing to organ dysgenesis, whereas heterozygous animals develop a spectrum of phenotypes, with hepatic hemangiomas, renal carcinoma and renal cysts being prevalent (Kobayashi et al., 2001; Kwiatkowski et al., 2002; Onda et al., 1999). Conditional *Tsc1-* and *Tsc2-*knockout animal models have also replicated some of the neuropathological features of TSC. Ablation of hamartin or tuberin in cerebellar Purkinje cells leads to an increase in soma size and dendritic spine density (Reith et al., 2013; Tsai et al., 2012). Moreover, loss of hamartin in the cerebral cortex and hippocampus leads to dysplastic neurons, ectopic neurons and reduced myelination, whereas astrocyte-specific deletion of *Tsc1* initiates astrogliosis and the aberrant migration of hippocampal pyramidal neurons (Meikle et al., 2007; Uhlmann et al., 2002). Such changes to CNS architecture subsequently lead to functional and autistic-like behavioural deficits (McMahon et al., 2014; Meikle et al., 2007; Reith et al., 2013; Tavazoie et al., 2005; Tsai et al., 2012; Uhlmann et al., 2002). However, although these previous conditional ablation studies have generated substantial insight into the neurological and behavioural aspects of TSC, it is still imperative to generate innovative models that specifically address the roles of hamartin and tuberin in other TSC-affected organs. This is especially true if animal models are to be used as platforms to preclinically evaluate novel therapeutic approaches for the treatment of this multi-organ disorder (Bissler et al., 2013; Franz et al., 2013; Napolioni et al., 2009; Samueli et al., 2015).

An animal model that addresses the involvement of the eye and visual system in TSC is currently overlooked. This is especially surprising because: (i) clinical examination of the eye is one of the original diagnostic procedures used to demonstrate CNS involvement in TSC, (ii) three distinct morphological groups of retinal hamartomas are routinely observed in individuals with TSC, and (iii) approximately 50% of all TSC-affected individuals present with eye involvement (Crino, 2013; Gomez, 1991; Mennel et al., 2007; Samueli et al., 2015; Sepp et al., 1996; Shields et al., 2004). We report here the generation and characterisation of an eye-specific TSC mouse model that recapitulates the classic neuropathological hallmarks of this syndrome, and also demonstrate a pivotal role for *Tsc1* in regulating various aspects of visual-pathway development. Our results provide the first major insight into the molecular etiology of TSC within the developing eye.

**TRANSLATIONAL IMPACT**

**Clinical issue**

Tuberous sclerosis complex (TSC) is a rare, inherited syndrome that is characterised by neurodevelopmental deficits and the presence of benign tumours, known as hamartomas, in affected organs. The disease is caused by mutations in either of two genes, *TSC1* or *TSC2*, which are involved in cell proliferation and differentiation. Mutations in these genes induce aberrant activation of the mammalian target of rapamycin complex 1 (mTORC1) pathway, leading to uncontrolled cell growth and tumour development. In addition to hamartomas commonly affecting the brain, kidney, skin and heart, lesions to the eye are central to TSC diagnosis. However, the consequences of TSC for the visual system are not known. Moreover, an animal model that can be used to specifically assess the involvement of the visual system in TSC is currently lacking.

**Results**

Here, the authors use a loss-of-function approach to generate a novel eye-specific TSC mouse model by conditional ablation of the *Tsc1* gene. Levels of hamartin, the protein encoded by *Tsc1*, were reduced specifically in the eyes upon ablation of the gene. The mutant mice recapitulate many of the neuropathological hallmarks of TSC, such as hamartoma-like lesions with retinal detachment, eye enlargement and loss of retinal architecture. The authors performed a detailed characterisation of the model at the cellular level, which included analysis by optical projection tomography, immunohistochemistry and scanning electron microscopy. These analyses revealed multiple defects in visual-pathway development, including aberrant neuronal differentiation, migration and circuit formation.

**Implications and future directions**

This work provides the first retina-specific model of TSC. The model strikingly mimics the neuropathological characteristics of the disease, validating its use in studies examining the pathogenic effects of *Tsc1* loss in the eye. Moreover, the authors provide the first major insight into the molecular etiology of TSC within the visual system, paving the way for a better understanding of the underlying retinal pathology. This novel model establishes a foundation for future studies that aim to predict clinical complications of TSC in the visual system. In addition, the model provides a platform for evaluating new therapeutic approaches for the treatment of this multi-organ disorder.

## RESULTS

### Generation of an eye-specific *Tsc1* conditional-knockout mouse model

**Fig. 1.**
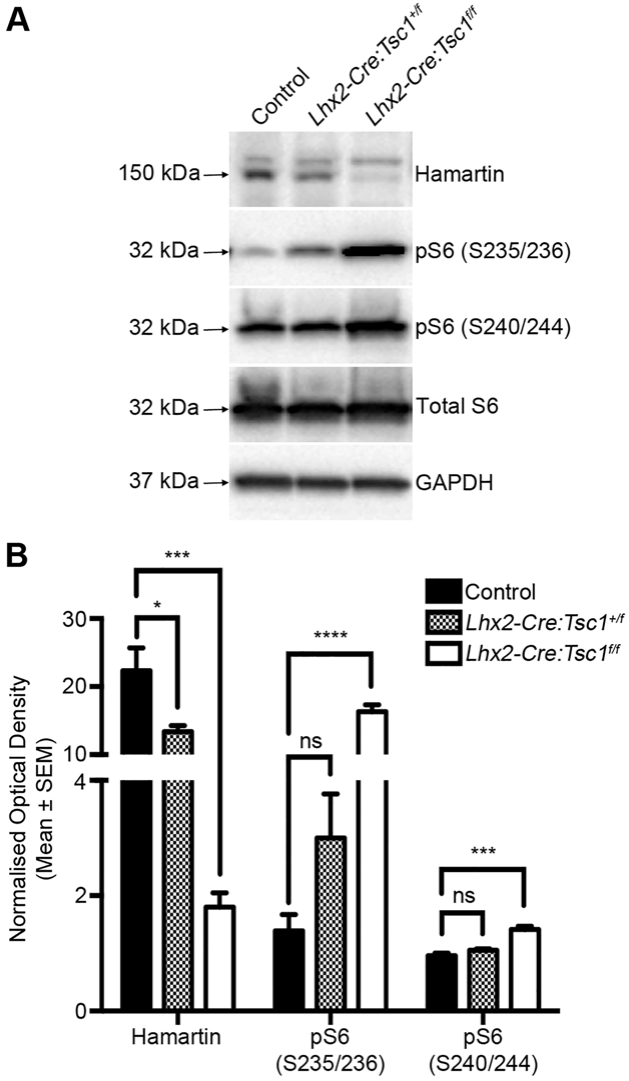
**Conditional deletion of *Tsc1* during eye development leads to an activation of the mTORC1 pathway.** (A) Immunoblot analysis of hamartin, total S6, pS6 (S235/236), pS6 (S240/244) and GAPDH from control, *Lhx2-Cre:Tsc1^+/f^* and *Lhx2-Cre:Tsc1^f/f^* eye extracts. (B) Densitometry analysis demonstrates that conditional deletion of *Tsc1* results in a reduction of hamartin level and a parallel upregulation in pS6 (S235/236) and pS6 (240/244) levels. GAPDH was used for normalization of hamartin densitometry data. Total S6 was used for normalization of pS6 (S235/235) and pS6 (S240/244) densitometry data. All data represent the mean±s.e.m. of three mice from each genotype. Abbreviations: GAPDH, glyceraldehyde 3-phosphate dehydrogenase; kDa, kilodalton. *P*-values are denoted as follows: ns, not significant, *P≤0.05, ***P≤0.001, ****P≤0.0001.

To generate an eye-specific TSC animal model, we used a loss-of-function approach by breeding mice carrying a conditional *Tsc1* allele (*Tsc1^tm1Djk^*; referred to as *Tsc1^+/f^* or *Tsc1^f/f^*) with mice harbouring an *Lhx2*-promoter-driven Cre-recombinase transgene [*Tg(Lhx2-Cre)1Lcar*; referred to as *Lhx2-Cre*] (Hägglund et al., 2011; Uhlmann et al., 2002). This transgene promotes recombination starting at embryonic day 8.25 (E8.25) and is expressed in eye-committed neural progenitor cells that give rise to the retinal pigment epithelium (RPE), neural retina (NR) and optic stalk (Fig. S1A) (Hägglund et al., 2011). To confirm deletion of *Tsc1*, we performed immunoblot analysis on eye homogenates (Fig. 1A). A significant reduction in the amount of hamartin was detected in both *Lhx2-Cre:Tsc1^+/f^* and *Lhx2-Cre:Tsc1^f/f^* mice compared with controls (*n*=3), suggesting that *Tsc1* was ablated in an eye-specific manner (Fig. 1B). A major cellular consequence of *Tsc1* loss is the activation of mTORC1 kinase complex and subsequent increase in the levels of phospho-S6 ribosomal protein (pS6) (Kwiatkowski et al., 2002; Meikle et al., 2007). We observed a significant increase in pS6 (S235/236) and pS6 (S240/244) levels in *Lhx2-Cre:Tsc1^f/f^* mice when compared to controls (Fig. 1A,B). Thus, conditional deletion of *Tsc1* during eye development results in a reduction of hamartin levels and subsequent hyperactivation of the mTORC1 pathway.

### Conditional deletion of *Tsc1* leads to enlarged eyes, hamartomas and loss of retinal architecture

**Fig. 2.**
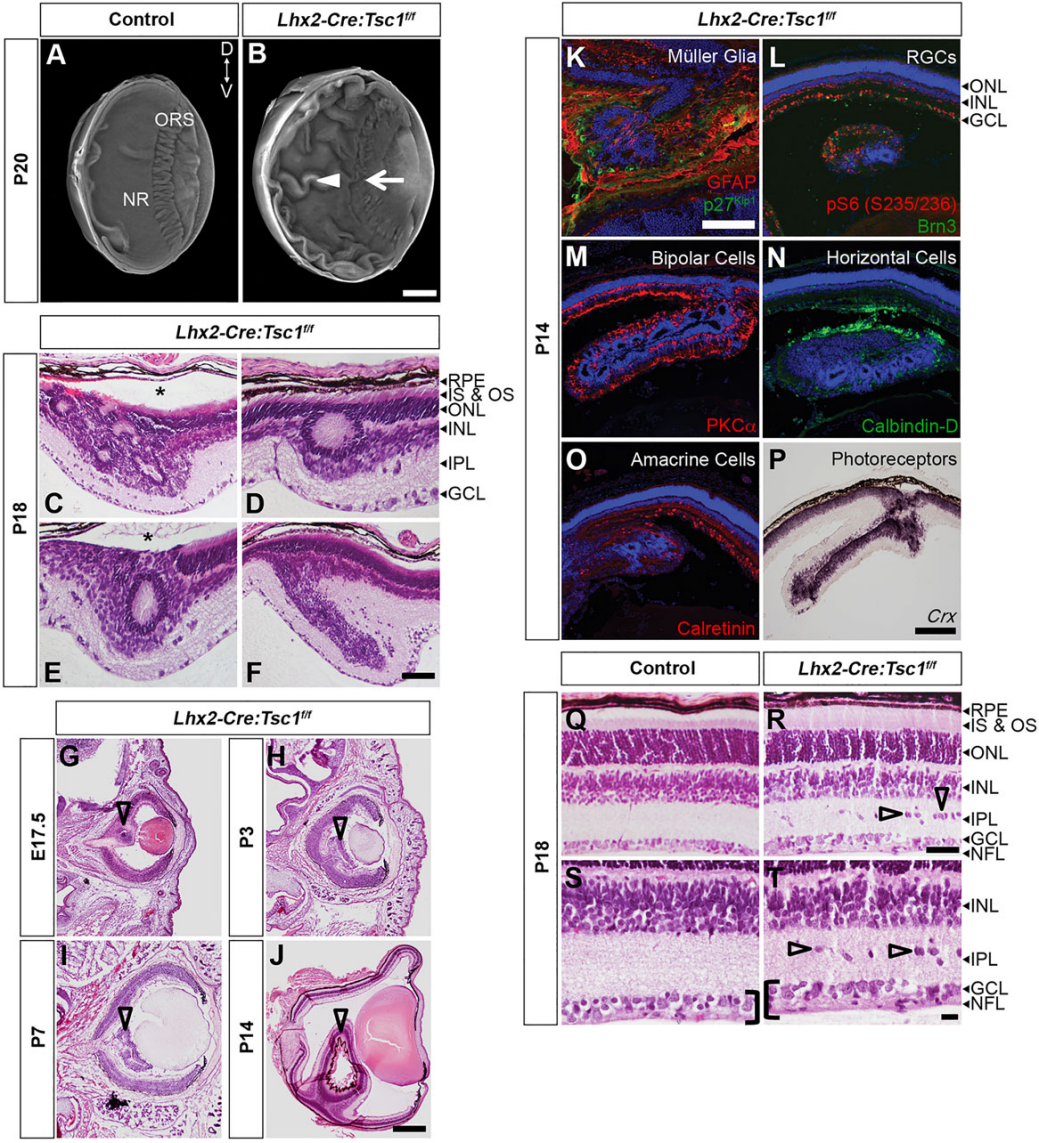
**Conditional deletion of *Tsc1* leads to enlarged eyes, hamartomas and loss of retinal architecture.** (A,B) OPT 3D volume rendering from control (A) and *Lhx2-Cre:Tsc1^f/f^* (B) mice, showing an enlargement of the eye, retinal folding (arrowhead) and loss of ora serrata (ORS) integrity (arrow) in *Lhx2-Cre:Tsc1^f/f^* mice. (C-F) Histological analysis of coronal eye sections demonstrating that the retinal folds observed during OPT analysis of *Lhx2-Cre:Tsc1^f/f^* mice are hamartomas that were organised into ring heterotopias. Moreover, retinal detachment was a common occurrence in areas of hamartoma formation (C,E, asterisks). (G-J) Histological analysis of coronal eye sections demonstrating that hamartomas first become evident during late embryogenesis (G, arrowhead) in *Lhx2-Cre:Tsc1^f/f^* mice. These lesions then become more pronounced during postnatal development (H-J, arrowheads). (K-P) Immunostaining and *in situ* hybridisation analyses demonstrating that hamartomas in *Lhx2-Cre:Tsc1^f/f^* mice are enriched in pS6 (S235/236) protein (L) and consist of all the cellular classes that populate the retina: Müller glia (K, GFAP^+^, p27^Kip1+^), RGCs (L, Brn3^+^), bipolar cells (M, PKCα^+^), horizontal cells (N, calbindin-D^+^), amacrine cells (O, calretinin^+^) and photoreceptors (P, *Crx^+^*). (Q-T) Histological analysis of coronal eye sections from control (Q,S) and *Lhx2-Cre:Tsc1^f/f^* (R,T) mice demonstrate the irregular ordering of the INL, an IPL populated with ectopic cells (R,T, arrowheads) and a widening of the GCL and NFL (S,T, brackets) in *Lhx2-Cre:Tsc1^f/f^* mice. Scale bars: (A,B,G-J) 500 µm; (C-F,Q-T) 50 µm; (K) 100 µm; (L-P) 200 µm. Abbreviations: Brn, brain-specific homeobox; *Crx*, cone-rod homeobox; D, dorsal; GCL, ganglion cell layer; GFAP, glial fibrillary acidic protein; IPL, inner plexiform layer; IS, inner segments; INL, inner nuclear layer; NFL, nerve fiber layer; NR, neural retina; ORS, ora serrata; OS, outer segments; PKC, protein kinase C; RPE, retinal pigment epithelium; V, ventral.

We first examined the morphology of the eye in *Lhx2-Cre:Tsc1^f/f^* mice, because hamartoma formation is one of the pathological hallmarks of TSC (Samueli et al., 2015). Optical projection tomography (OPT) analysis demonstrated that the eye of postnatal *Lhx2-Cre:Tsc1^f/f^* mice was enlarged when compared to control animals (Fig. 2A,B). Most striking, however, was the presence of severe retinal folding (Fig. 2B, white arrowhead) and the loss of ora serrata (ORS) integrity (Fig. 2B, white arrow). These observations indicated that *Tsc1* ablation had a profound impact upon retinal morphology and that *Lhx2-Cre:Tsc1^f/f^* mice were therefore a plausible model to assess eye involvement in TSC.

The adult retina contains one glial cell type (Müller glia) and six classes of neurons (rod and cone photoreceptors, and horizontal, bipolar, amacrine and ganglion cells) whose cell bodies reside within three distinct layers: photoreceptor cell bodies reside in the outer nuclear layer (ONL); the inner nuclear layer (INL) contains horizontal, bipolar, amacrine and Müller glia cell bodies; and the ganglion cell layer (GCL) contains ganglion cells, whose axons form the nerve fiber layer (NFL), and displaced amacrine cells. Synapses between photoreceptors and bipolar and horizontal cells occur in the outer plexiform layer (OPL), whereas connections between bipolar, amacrine and ganglion cells occur in the inner plexiform layer (IPL) (Fig. 2Q) (Bassett and Wallace, 2012; Livesey and Cepko, 2001).

Histological staining of coronal eye sections demonstrated that the retinal folding observed in the OPT-derived three-dimensional (3D) volumes were hamartomas (Fig. 2C-F), which were commonly organised into ring heterotopias (Fig. 2C-E), with retinal detachment also being occasionally observed in areas of hamartoma formation (Fig. 2C,E, asterisks). The hamartomas were subsequently determined to arise during late embryogenesis and were first consistently detectable at E17.5 (Fig. 2G). Moreover, these lesions were unlikely to arise as a secondary consequence to aberrant proliferation at the early embryonic ages we analysed because the percentage of BrdU^+^ cells in the neural retina of control and *Lhx2-Cre:Tsc1^f/f^* mice did not differ (Fig. S2).

The hamartomas then became more pronounced during postnatal development (Fig. 2H-J) and consisted of all the cellular classes that are contained within the retina. In addition, the hamartomas were also enriched in pS6 (S235/236) and contained elevated levels of glial fibrillary acidic protein (GFAP), which is indicative of reactive gliosis (Fig. 2K-P). Finally, although laminar organisation of the retina in *Lhx2-Cre:Tsc1^f/f^* mice was grossly maintained (Fig. 2Q-T), several other abnormalities were readily apparent, including an irregular ordering of the INL and an IPL populated with ectopic cells (Fig. 2R,T, arrowheads). We also observed a widening of the GCL and NFL (Fig. 2S,T, black brackets). This was corroborated by neurofilament (NF) immunostaining, which demonstrated disorganised nuclei and aberrant neurite trajectories in the GCL and NFL of *Lhx2-Cre:Tsc1^f/f^* mice (Fig. S3).

### Conditional deletion of *Tsc1* leads to aberrant neurite stratification of retinal interneurons

**Fig. 3.**
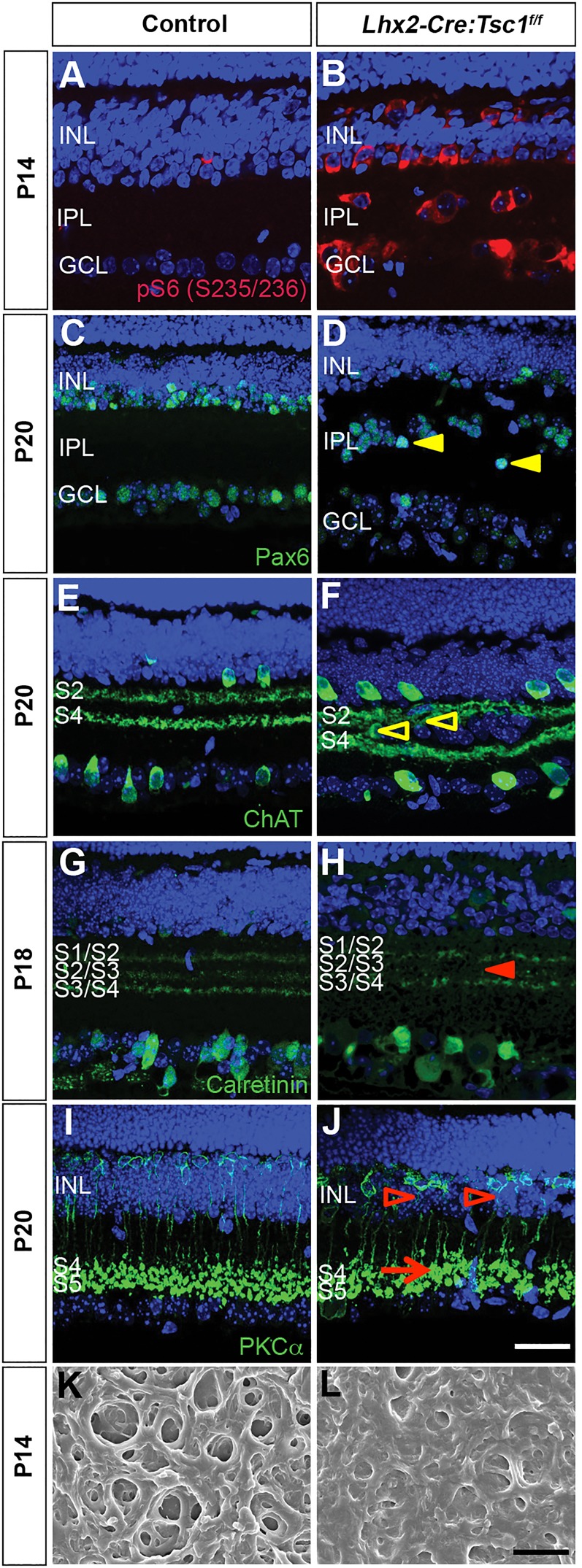
**Conditional deletion of *Tsc1* leads to aberrant neurite stratification of retinal interneurons.** (A,B) Coronal eye sections from control (A) and *Lhx2-Cre:Tsc1^f/f^* (B) mice showing global upregulation of pS6 (S235/236) in the INL and GCL of *Lhx2-Cre:Tsc1^f/f^* mice. (C-J) Coronal retinal sections from control (C,E,G,I) and *Lhx2-Cre:Tsc1^f/f^* (D,F,H,J) mice. Amacrine cells (D, Pax6^+^) have irregular cell-body positions in the INL of *Lhx2-Cre:Tsc1^f/f^* mice. Also evident is the presence of ectopic amacrine cells or RGCs (Pax6^+^) in the IPL (D, arrowheads). Stratification deficits were observed in the *Lhx2-Cre:Tsc1^f/f^* mice that ranged from ectopic neurites extending into other sublaminae (F, ChAT^+^, arrowheads) to a loss of specific sublamina stratification (H, calretinin^+^, arrowhead). Moreover, rod bipolar cells in *Lhx2-Cre:Tsc1^f/f^* mice have distorted axonal projections that extend through the INL (J, PKCα^+^, arrowheads) and terminate with enlarged axon pedicles in the IPL (J, PKCα^+^, arrow). (K,L) Scanning electron microscopic (SEM) analysis of the IPL in control (K) and *Lhx2-Cre:Tsc1^f/f^* (L) mice. The density of the IPL in *Lhx2-Cre:Tsc1^f/f^* mice was increased when compared to control mice. Scale bars: (A-J) 25 µm; (K-L) 1 µm. Abbreviations: ChAT, choline acetyltransferase; GCL, ganglion cell layer; IPL, inner plexiform layer; INL, inner nuclear layer; Pax, paired box protein; PKC, protein kinase C; S1-S5, sublaminae 1-5.

Conditional deletion of *Tsc1* in the eye led to several morphological changes within the retina that recapitulated many of the hallmarks of TSC. We therefore took advantage of this eye-specific model to determine whether the laminar disorganisation observed in *Lhx2-Cre:Tsc1^f/f^* mice influenced neuronal circuitry. We first chose to focus on amacrine and bipolar neurons because we observed a disorganised INL and IPL that contained cells with elevated levels of pS6 (S235/236) protein in *Lhx2-Cre:Tsc1^f/f^* mice (Fig. 3A,B). The IPL is subdivided into two parallel circuits that respond to either a decrease (OFF pathway) or an increase (ON pathway) in light intensity. These circuits are organised into distinct sublaminae (S) that allow for the processing of visual information (OFF sublaminae: S1 and S2; ON sublaminae: S3, S4 and S5) (Sanes and Zipursky, 2010).

To identify all amacrine cells, we analysed for the presence of Pax6 protein (Ma et al., 2007). The amacrine cell layer (Pax6^+^) was severely disrupted within the INL of *Lhx2-Cre:Tsc1^f/f^* mice (Fig. 3C,D). A high number of Pax6^+^ cells were also observed within the IPL, suggesting that the majority of ectopic cells observed in our mutant mice are either amacrine or retinal ganglion cells (RGCs) because this neuron class is also recognised by the anti-Pax6 antibody (Fig. 3D, yellow arrowheads) (Ma et al., 2007). To investigate whether the disorganised cell-body layers influenced neurite positioning within the IPL, we analysed the stratification patterns of cholinergic (ChAT^+^, S2 and S4) and calretinin^+^ (S1/S2, S2/S3 and S3/S4) amacrine cell subtypes (Fig. 3E-H). Ectopic ChAT^+^ neurites could be clearly seen extending into other sublaminae (Fig. 3F, arrowheads), whereas a loss of specific sublamina stratification (e.g. S2/S3) was observed for the calretinin^+^ cells (Fig. 3H, arrowhead). Moreover, rod bipolar cells (PKCα^+^) elaborated distorted axonal projections through the INL (Fig. 3J, arrowheads) and terminated with hypertrophic axon pedicles within enlarged S4 and S5 ON sublaminae (Fig. 3J, arrow). The ectopic-cell and dendritic-stratification phenotype had full penetrance: we observed at least one region in each eye analysed (*n*=18) that contained ectopic cells, ectopic neurites or loss of stratification. The fact that we observed ectopic Pax6^+^ cells, aberrant neurite stratification and changes to sublaminae formation suggests profound changes to the structure of the IPL in *Lhx2-Cre:Tsc1^f/f^* mice. We therefore performed scanning electron microscopy (SEM) and observed that the IPL in *Lhx2-Cre:Tsc1^f/f^* mice was overgrown and densely packed when compared to control animals (Fig. 3K,L). In conclusion, our observations demonstrate that *Tsc1* is involved in regulating cell-body position within the INL and stratification within the IPL. Moreover, the overgrown and densely packed IPL might form a physical barrier that could partly explain the presence of ectopic cells within this region.

### Conditional deletion of *Tsc1* leads to accelerated RGC differentiation and radial-migration defects

Our immunostaining analyses demonstrated that the disorganised cells in the GCL of *Lhx2-Cre:Tsc1^f/f^* mice were enriched in pS6 (S235/236) protein (Fig. 3B). Moreover, aberrant and defasciculated axonal projections were also evident in the NFL (Fig. S3B). These combined observations suggest that *Tsc1* is involved in regulating several aspects of RGC biology. We therefore chose to examine RGC differentiation in our TSC mouse because this cell type is the first neuronal class to differentiate in the retina and they appear following *Tsc1* ablation. RGCs are also an ideal model in which to study visual-pathway development from the perspectives of both neuronal circuitry and axonal guidance (Erskine and Herrera, 2007). Firstly, we examined RGC differentiation during embryonic and postnatal ages and chose time points that coincided with the important milestones of RGC development: neurogenesis (E12.5), migration (E17.5) and target innervation [postnatal day 1 (P1) through P7] (Huberman et al., 2008; Reese, 2011).

**Fig. 4.**
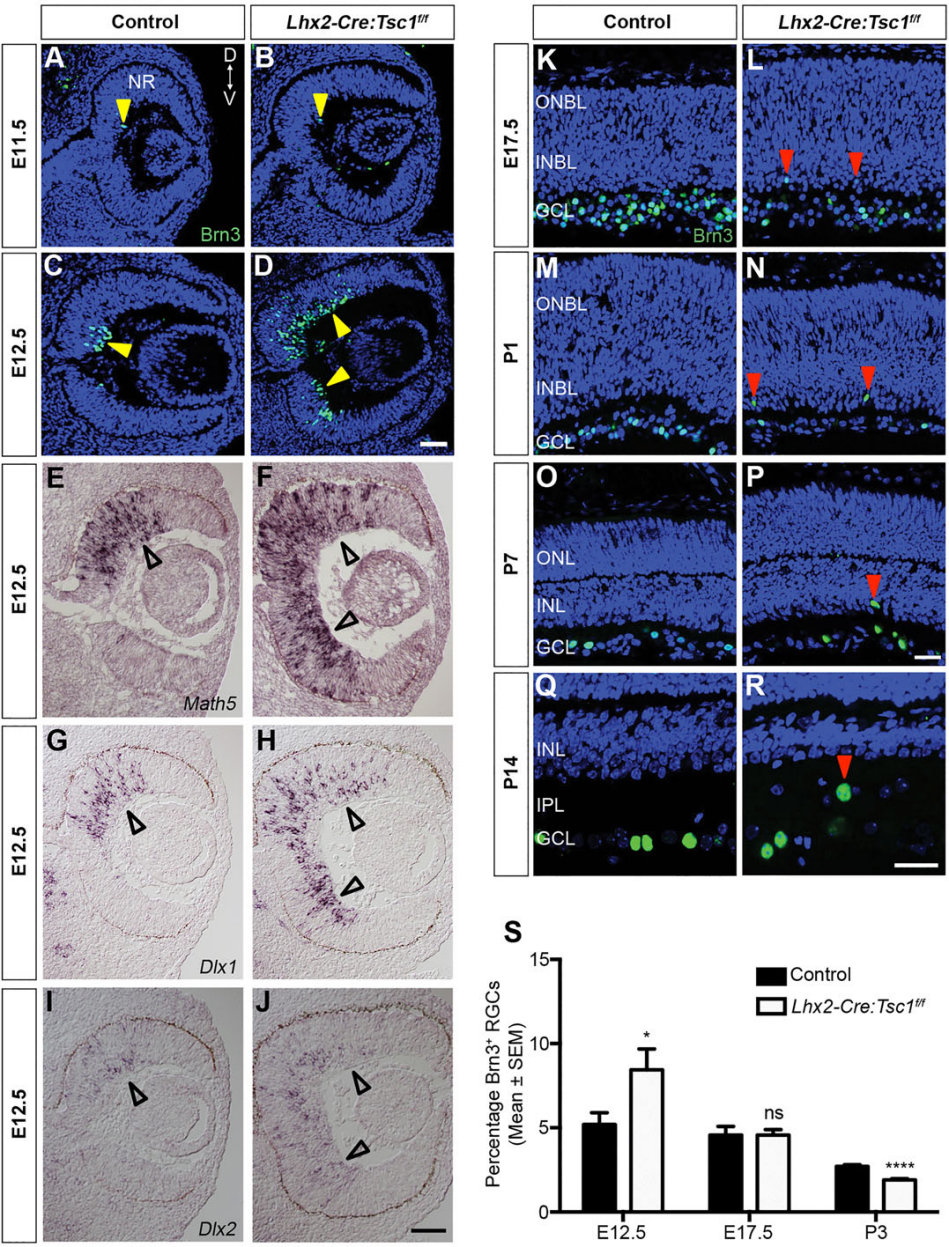
**Conditional deletion of *Tsc1* leads to accelerated RGC differentiation and radial-migration defects*.*** (A-D) Immunostaining analysis of Brn3^+^ RGCs (arrowheads) in control and *Lhx2-Cre:Tsc1^f/f^* mice at E11.5 (A,B) and E12.5 (C,D) demonstrates the accelerated appearance of RGCs in *Lhx2-Cre:Tsc1^f/f^* mice. (E-J) *In situ* hybridisation analysis at E12.5 demonstrates expanded expression domains for *Math5* (E,F), *Dlx1* (G,H) and *Dlx2* (I,J) in *Lhx2-Cre:Tsc1^f/f^* mice (arrowheads). (K-R) Spatiotemporal analysis of RGC position during migration (E17.5, K,L), target innervation (P1, M,N and P7, O,P) and refinement (P14, Q,R) demonstrates that RGCs radially migrate to reach their final laminar position within the GCL by early postnatal ages in control mice (K,M,O,Q). In contrast, ectopic RGCs begin to appear at E17.5 (L, arrowheads) and become more pronounced after birth in *Lhx2-Cre:Tsc1^f/f^* mice (N,P,R, arrowheads). (S) Quantification of Brn3^+^ RGC number in control and *Lhx2-Cre:Tsc1^f/f^* mice during neurogenesis (E12.5), migration (E17.5), and target innervation and synapse formation (P3). Although the initial numbers of Brn3^+^ RGCs was increased in the mutant mice (E12.5), there is a subsequent reduction in the number of Brn3^+^ cells that continues through late embryonic (E17.5) and early postnatal (P3) development. The data represent the mean±s.e.m. of at least three mice from each genotype. Scale bars: (A-D;E-J) 50 µm; (K-P;Q,R) 25 µm. Abbreviations: Brn, brain-specific homeobox; D, dorsal; *Dlx*, distal-less homeobox; GCL, ganglion cell layer; INBL, inner neuroblastic layer; *Math*, mouse atonal; NR, neural retina; ONBL, outer neuroblastic layer; RGC, retinal ganglion cell; V, ventral. *P*-values are denoted as follows: ns, not significant, *P≤0.05, ****P≤0.0001 compared with controls.

To follow RGC differentiation, we analysed for the presence of Brn3 protein (Pan et al., 2005). Initially, we found a few scattered Brn3^+^ RGCs at E11.5 in both control and *Lhx2-Cre:Tsc1^f/f^* mice (Fig. 4A,B, arrowheads). The initial wave of RGCs born in the dorsocentral retina was detected at E12.5 in control mice (Fig. 4C, arrowhead). However, we found an increase in the number of Brn3^+^ cells in the *Lhx2-Cre:Tsc1^f/f^* mice that were dispersed both dorsally and ventrally throughout the NR (Fig. 4D, arrowheads). To confirm this accelerated differentiation, we analysed the expression patterns of *Math5*, *Dlx1* and *Dlx2*, because these genes are essential for RGC development (de Melo et al., 2003; Yang et al., 2003). Whereas the expression of *Math5*, *Dlx1* and *Dlx2* in control mice was observed only in the dorsocentral retina (Fig. 4E,G,I, arrowheads), the expression domains of these genes in *Lhx2-Cre:Tsc1^f/f^* mice were expanded both dorsally and ventrally into the peripheral NR, as was observed for Brn3 (Fig. 4F,H,J, arrowheads). Moreover, the maintenance of dorsoventral identity based on the expression domains of *Tbx5* and *Vax2* demonstrates that the accelerated differentiation of RGCs in the *Lhx2-Cre:Tsc1^f/f^* mice was not due to changes in dorsoventral patterning (Fig. S4) (Behesti et al., 2006).

RGC differentiation in control mice proceeded in a wave from central to peripheral retina and, by E17.5, all the Brn3^+^ cells had migrated into their final laminar position within the future GCL (Fig. 4K). The GCL then became more defined during the first postnatal week (P1, Fig. 4M and P7, Fig. 4O) and, by the completion of neurogenesis at P14, it contained an ordered radial distribution of Brn3^+^ RGCs (Fig. 4Q) (Young, 1985). In contrast, although the majority of Brn3^+^ cells in the *Lhx2-Cre:Tsc1^f/f^* mice were observed to migrate into their final laminar position, ectopic cells were detected beginning at E17.5 (Fig. 4L, arrowheads), and these cells became readily apparent in all mice analysed at postnatal ages (P1, Fig. 4N; P7, Fig. 4P; P14, Fig. 4R; red arrowheads). Thus, conditional deletion of *Tsc1* induces accelerated differentiation and aberrant radial migration of RGCs.

### Conditional deletion of *Tsc1* leads to a reduction in RGC number with the remaining cells exhibiting enlarged nuclei and an asymmetric mosaic distribution

**Fig. 5.**
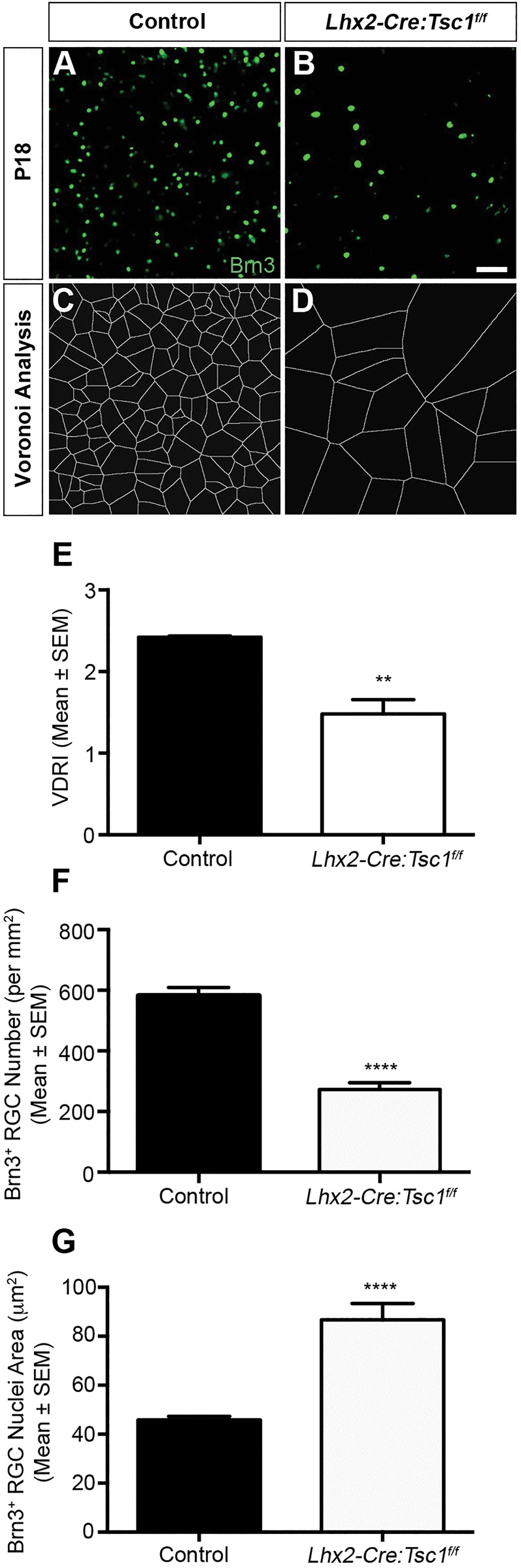
**Conditional deletion of *Tsc1* leads to a reduction in RGC number and asymmetric mosaic distribution.** (A,B) Retinal flat-mount analysis of Brn3^+^ RGCs in the tangential plane of control (A) and *Lhx2-Cre:Tsc1^f/f^* (B) mice shows a reduction in the number of RGCs in *Lhx2-Cre:Tsc1^f/f^* mice. (C,D) Voronoi domain analysis of the immunostaining images demonstrates an increase in cell territories occupied in *Lhx2-Cre:Tsc1^f/f^* mice. (E) Voronoi domain regularity indices for control and *Lhx2-Cre:Tsc1^f/f^* mice. A reduction in mosaic regularity was observed for *Lhx2-Cre:Tsc1^f/f^* mice. The data represent the mean±s.e.m. of three mice from each genotype. (F) Quantification of RGC number in the tangential plane of control and *Lhx2-Cre:Tsc1^f/f^* mice. A decrease in the number of Brn3^+^ cells was observed in *Lhx2-Cre:Tsc1^f/f^* mice. The data represent the mean±s.e.m. of three mice from each genotype. (G) Quantification of the cross-sectional RGC nucleus area in the tangential plane of control and *Lhx2-Cre:Tsc1^f/f^* mice. An increase in the cross-sectional area of Brn3^+^ cells was observed in *Lhx2-Cre:Tsc1^f/f^* mice. The data represent the mean±s.e.m. of three mice from each genotype. Scale bar: (A,B) 50 µm. Abbreviations: Brn, brain-specific homeobox; RGC, retinal ganglion cell; VDRI, Voronoi domain regularity index. *P*-values are denoted as follows: **P≤0.01, ****P≤0.0001 compared with controls.

The proportion of Brn3^+^ RGCs seemed to be reduced in postnatal *Lhx2-Cre:Tsc1^f/f^* mice. This was confirmed by cell count analysis (Fig. 4S). Although the initial numbers of Brn3^+^ RGCs was increased in *Lhx2-Cre:Tsc1^f/f^* mice due to accelerated differentiation (E12.5), there was a subsequent reduction in the population of Brn3^+^ cells through embryonic (E17.5) and postnatal (P3) development compared to control animals. We therefore performed flat-mount analyses in order to further quantify the reduction in RGC numbers following the completion of retinal neurogenesis (Fig. 5A,B) (Young, 1985). In control retinas, Brn3^+^ nuclei were dispersed in regularly patterned mosaics. By contrast, loss of RGC nuclei in *Lhx2-Cre:Tsc1^f/f^* mice resulted in asymmetric mosaic distribution. To confirm this irregularity, we examined the spatial properties using Voronoi analysis, which computes the territory surrounding each cell relative to its neighbours (Cantrup et al., 2012; Galli-Resta et al., 1997; Kay et al., 2012). The Voronoi domain diagrams revealed an increase in cell territory occupied in *Lhx2-Cre:Tsc1^f/f^* mice, whereas the Voronoi regularity index demonstrated that these enlarged territories were organised as irregular mosaics (Fig. 5C-E). To confirm RGC loss, we performed cell-count analysis and observed a significant reduction in the number of Brn3^+^ cells in *Lhx2-Cre:Tsc1^f/f^* mice compared to controls (Fig. 5F). We reasoned that an increased rate of apoptosis could account for this observation and therefore quantified the number of activated-caspase3^+^ cells on serial sections taken from comparable regions of control and *Lhx2-Cre:Tsc1^f/f^* retinas. At all ages analysed, we observed a significant increase in the number of apoptotic cells in *Lhx2-Cre:Tsc1^f/f^* mice (Fig. S5). Our observations therefore demonstrate that an increased level of programmed cell death is one mechanism that contributes to the decreased numbers of RGCs in *Lhx2-Cre:Tsc1^f/f^* mice. Finally, the remaining RGC nuclei seemed to be enlarged in *Lhx2-Cre:Tsc1^f/f^* mice. To quantify this enlargement, we measured the cross-sectional area of Brn3^+^ cells in the same retinal flat mounts. We observed a significant increase in the size of the remaining RGC nuclei within *Lhx2-Cre:Tsc1^f/f^* mice compared to controls (Fig. 5G). In summary, conditional deletion of *Tsc1* leads to a loss of RGCs at postnatal ages and this reduction in cell number leads to asymmetric mosaics in *Lhx2-Cre:Tsc1^f/f^* mice. Of the RGCs that survive, these cells exhibit increased nuclei size. *Tsc1* is therefore critical for regulating cell survival and size.

### Conditional deletion of *Tsc1* leads to a loss of retinogeniculate topography

**Fig. 6.**
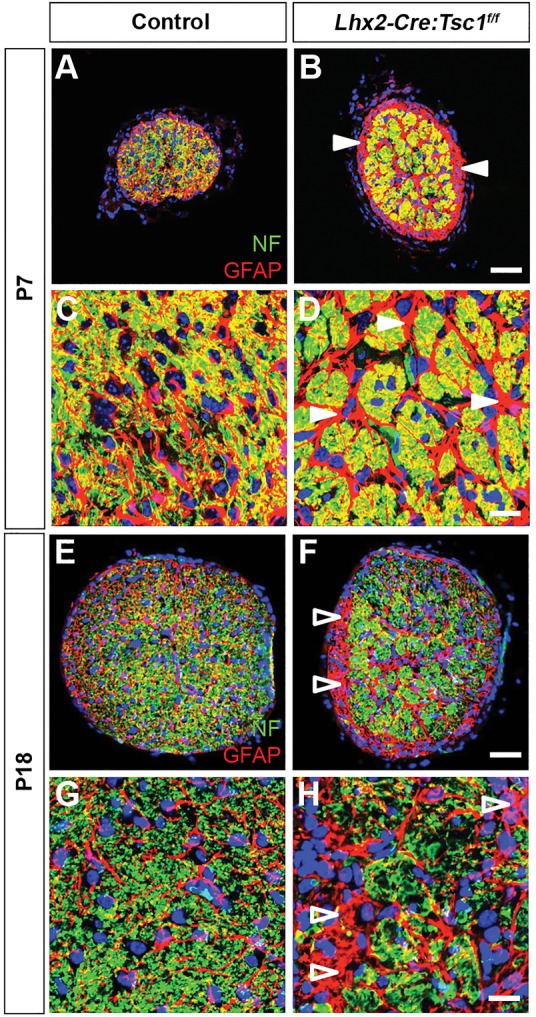
**Conditional deletion of *Tsc1* leads to reactive gliosis and changes in optic nerve morphology.** (A-D) Transverse sections of the ON at P7 demonstrate a defined network of GFAP^+^ astrocytes that enmesh the NF^+^ RGC axons in control mice (A,C). In contrast, regionalised areas of reactive gliosis are clearly visible in the ON of *Lhx2-Cre:Tsc1^f/f^* mice (B,D, arrowheads). (E-H) Transverse sections of the ON at P18 demonstrate a defined network of GFAP^+^ astrocytes that enmesh the NF^+^ RGC axons in control mice (E,G). In contrast, axon loss is clearly visible in regionalised areas of the ON from *Lhx2-Cre:Tsc1^f/f^* mice. Also evident are cross-sectional areas that are significantly enriched in GFAP^+^ astrocytes and completely devoid of NF^+^ RGC axons (F,H, arrowheads). Scale bars: (A,B;E,F) 50 µm; (C,D;G,H) 25 µm. Abbreviations: ON, optic nerve; GFAP, glial fibrillary acidic protein; NF, neurofilament; RGC, retinal ganglion cell.

RGCs project their axons into the NFL, and these axons subsequently elaborate in a radial fashion to exit the eye at the optic disc. From here, they enter the optic nerves (ONs) and project through the optic tracts (OTs) to target the visual centres of the brain, including the dorsal lateral geniculate nucleus (dLGN) of the thalamus (Erskine and Herrera, 2007; Godement et al., 1984). Analysis of RGC axon trajectory during embryonic development demonstrated a comparable optic disc exit pattern in control and *Lhx2-Cre:Tsc1^f/f^* mice (Fig. S6). We therefore reasoned that changes in ON composition would be observed during postnatal development in *Lhx2-Cre:Tsc1^f/f^* mice because this time frame coincided with the greatest loss of RGC nuclei within the retina (Figs 4S, 5F and Fig. S5I). Transverse sections through the ON of control mice demonstrated a clearly defined network of GFAP^+^ astrocytes that enmeshed the neurofilament-positive (NF^+^) RGC axons within the ON at both P7 and P18 (Fig. 6A,C,E,G). In contrast, regionalised areas of reactive gliosis were clearly visible in several domains of the *Lhx2-Cre:Tsc1^f/f^* ON at P7 (Fig. 6B,D, arrowheads). Reactive gliosis in addition to regionalised axonal loss became more apparent at P18 in the *Lhx2-Cre:Tsc1^f/f^* mice: we observed cross-sectional areas that were significantly enriched in GFAP^+^ astrocytes and completely devoid of NF^+^ RGC axons (Fig. 6F,H, arrowheads). That these changes were observed as patches and not randomly distributed across the ON clearly correlates with the selective loss of RGC nuclei in discrete domains across the retina observed in our flat-mount analysis (Fig. 5B). These changes observed in the ON of *Lhx2-Cre:Tsc1^f/f^* mice were reminiscent to those documented in a *DBA/2J* mouse model of glaucoma (Howell et al., 2007). We therefore measured the intra ocular pressure (IOP) in control and *Lhx2-Cre:Tsc1^f/f^* mice and found them to be comparable (mean±s.e.m. of 18±0.82 and 21±1.9 mmHg for control and *Lhx2-Cre:Tsc1^f/f^* mice, respectively).

**Fig. 7.**
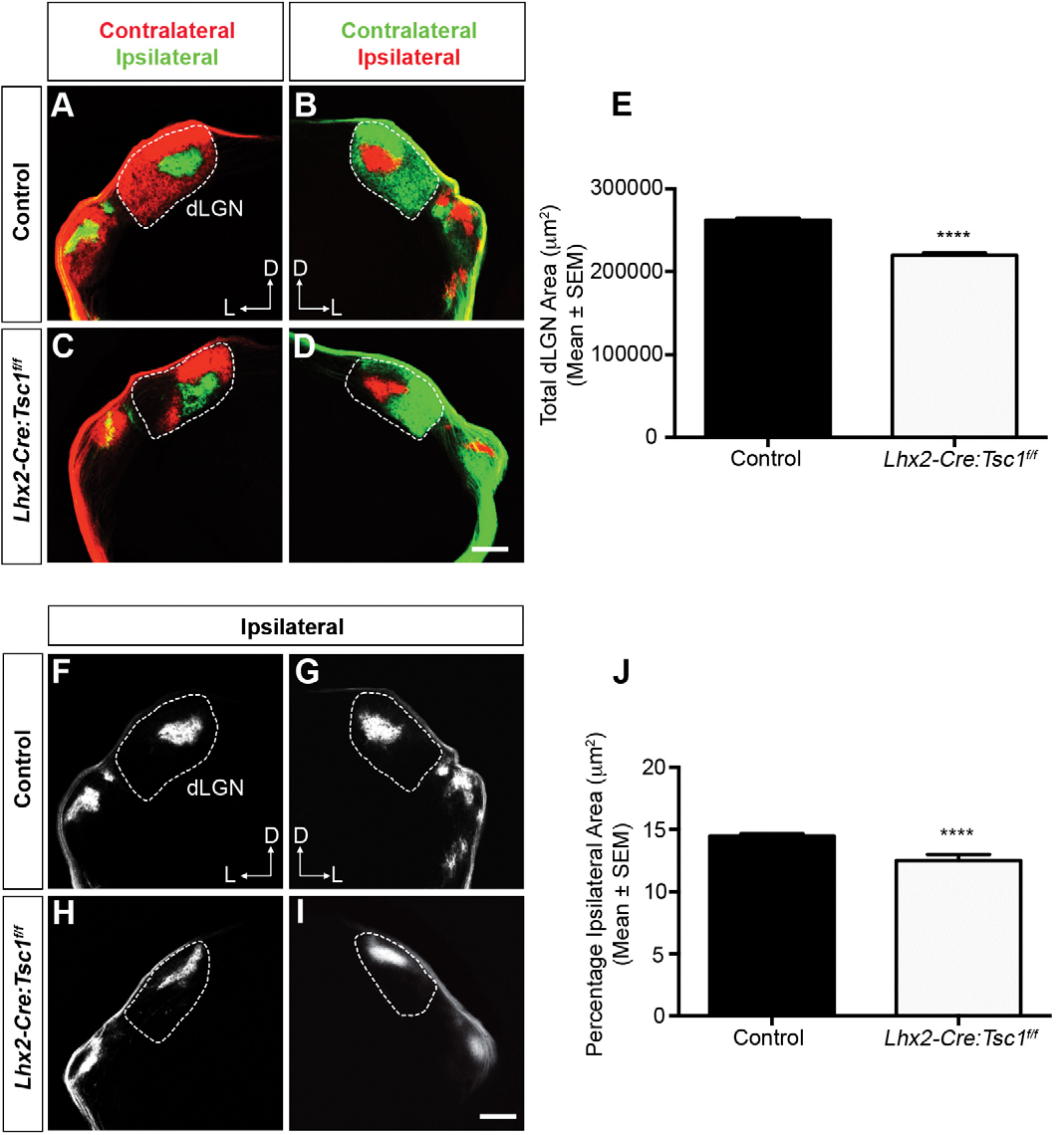
**Conditional deletion of *Tsc1* leads to aberrant retinogeniculate topography.** (A-D) Medial coronal sections showing retinogeniculate projections into the dLGN at P14 in control (A,B) and *Lhx2-Cre:Tsc1^f/f^* (C,D) mice. Dashed lines represent the borders of the dLGN. A reduction in the area occupied by both contralateral and ipsilateral projections is evident in *Lhx2-Cre:Tsc1^f/f^* mice. (E) Quantification of the total dLGN area (µm^2^) in control and *Lhx2-Cre:Tsc1^f/f^* mice at P14. The data represent the mean±s.e.m. of three mice from each genotype. A significant reduction in the total area occupied by both contralateral and ipsilateral projections is evident in *Lhx2-Cre:Tsc1^f/f^* mice. (F-I) Medial coronal sections showing the ipsilateral retinogeniculate projections into the dLGN at P14 in control (F,G) and *Lhx2-Cre:Tsc1^f/f^* (H,I) mice. Dashed lines represent the borders of the dLGN. The topographical appearance and position of the ipsilateral patch within the dLGN of *Lhx2-Cre:Tsc1^f/f^* mice was altered along the dorsoventral and lateromedial axis. (J) Quantification of the percentage ipsilateral projection area in control and *Lhx2-Cre:Tsc1^f/f^* mice at P14. The data represent the mean±s.e.m. of three mice from each genotype. A significant reduction in the percentage area occupied by the ipsilateral projection is evident in *Lhx2-Cre:Tsc1^f/f^* mice. Scale bars: (A-D,F-I) 250 µm. Abbreviations: D, dorsal; dLGN, dorsal lateral geniculate nucleus; L, lateral. *P*-values are denoted as follows: ****P≤0.0001 compared with controls.

We next proceeded to investigate whether the remaining axons in *Lhx2-Cre:Tsc1^f/f^* mice would target the dLGN. RGC projections were labelled with an anterograde tracer to visualize the binocular retinogeniculate projections within the dLGN (Fig. S7A). The inputs from each eye in control mice were segregated with a single ipsilateral patch in the dorsomedial quadrant of the dLGN being surrounded by the contralateral projection (Fig. 7A,B). As expected, a significant reduction in the area occupied by both contralateral and ipsilateral projections was observed in *Lhx2-Cre:Tsc1^f/f^* mice (Fig. 7C-E). This result clearly correlates with the dramatic reduction in total RGC number and regionalised axonal loss within the ON. We also observed a significant decrease in the percentage of the dLGN occupied by ipsilateral projections in *Lhx2-Cre:Tsc1^f/f^* mice (Fig. 7F-J). Also of note was the observation that the topography and position of the ipsilateral patch within the dLGN of *Lhx2-Cre:Tsc1^f/f^* mice was altered in all animals along the dorsoventral and lateromedial axis (Fig. 7H,I) and, in some instances, we observed more than one ipsilateral patch (Fig. S7B-E, yellow arrows). Our data therefore demonstrates that conditional deletion of *Tsc1* has a profound influence on the development of the visual pathway as demonstrated by a decrease in occupied contra- and ipsilateral territories within the dLGN.

## DISCUSSION

This study describes the generation of a novel eye-specific model of TSC that recapitulates many of the neuropathological hallmarks of this multi-organ disorder: increase in organ and cell size, ring heterotopias, hamartomas with retinal detachment, and lamination defects (Crino, 2013; Shields et al., 2004). Moreover, conditional ablation of *Tsc1* led to severe disruptions in visual-pathway development as evidenced by: (i) accelerated differentiation of RGCs, (ii) ectopic radial migration, (iii) neurite stratification deficits, (iv) an increase in apoptosis, (v) ON degeneration and (vi) aberrant retinogeniculate topography (Fig. S8).

The mutant mice described in this report differ substantially from the majority of published TSC models because we ablated *Tsc1* during early embryonic development in a progenitor cell population that generates both neural and glial lineages within the retina. This is in contrast to previous studies where the *Tsc1* or *Tsc2* genes have been ablated during later embryonic development in either neuronal or glial lineages independently (Ehninger et al., 2008; Feliciano et al., 2012; Meikle et al., 2007; Uhlmann et al., 2002; Way et al., 2009; Zeng et al., 2011; Zhou et al., 2011). Although these previous models documented some of the neuropathological changes associated with TSC, none of them recorded the presence of hamartoma-like lesions. This is presumably due to the fact that Cre-mediated recombination was initiated after tissue architecture was already established. We therefore propose that hamartoma formation occurs owing to the fact that the *Tsc1^tm1Djk^* allele is recombined in a progenitor cell population prior to the formation of any rudimentary eye structure (Hägglund et al., 2011) and that this might also be a potential mechanism that drives hamartoma formation in individuals with TSC. Although, it must be noted that our fate-mapping experiments demonstrated that the *Lhx2-Cre* transgene did not recombine the *ROSA26* allele in all parts of the retina in postnatal mice (Fig. S1B). We therefore concede that some of the hamartomas formed in *Lhx2-Cre:Tsc1^f/f^* mice could be mosaic in nature and composed of both *Tsc1*-null and normal cells. Non-cell-autonomous mechanisms could therefore also potentially contribute to hamartoma formation in our TSC model, as was previously demonstrated in zebrafish (Kim et al., 2011). We also cannot exclude the possibility that reactive Müller glia cells are also involved in hamartoma formation in *Lhx2-Cre:Tsc1^f/f^* mice, as was recently demonstrated for other rodent models of retinal degeneration (Hippert et al., 2015).

The eyes of *Lhx2-Cre:Tsc1^f/f^* mice were grossly indistinguishable from their littermates at birth. The developmental processes involved in the formation of the rudimentary eye structure, i.e. diencephalon evagination, optic vesicle formation, optic cup transformation and optic fissure closing, are therefore presumably independent of TSC-mTORC1 function (Adler and Canto-Soler, 2007). OPT analysis demonstrated that the initial striking feature of *Lhx2-Cre:Tsc1^f/f^* mice was that their eyes were enlarged compared to control littermates. Our observations corroborate the original *Drosophila melanogaster* studies in which an enlarged-eye phenotype was generated upon *Tsc1* or *Tsc2* ablation, and further demonstrate that mTORC1 signalling controls organ size within the nervous system (Anderl et al., 2011; Ehninger et al., 2008; Goto et al., 2011; Ito and Rubin, 1999; Magri et al., 2011; Potter et al., 2001; Tapon et al., 2001; Uhlmann et al., 2002; Way et al., 2009; Zeng et al., 2011; Zhou et al., 2011). Histological analysis demonstrated the presence of ectopic cells in the IPL and adds further support to the notion that TSC should be regarded as a neuronal migration syndrome (Feliciano et al., 2012, 2011; Gomez, 1991; Magri et al., 2011; Way et al., 2009). Ectopic cells within the IPL have also been observed upon deletion of other tumour suppressors such as *Zac1* and *PTEN* (Cantrup et al., 2012; Ma et al., 2007; Sakagami et al., 2012). The authors postulated that both *Zac1* and *PTEN* directly regulated cell migration by controlling the expression of cell adhesion genes (Cantrup et al., 2012; Ma et al., 2007; Varrault et al., 2006). That the loss of hamartin has also been documented to disrupt cell adhesion therefore provides a potential mechanism for the aberrant radial migration and the appearance of ectopic cells observed in this study (Lamb et al., 2000).

Neurites are segregated into distinct synaptic sublaminae within the IPL of the vertebrate retina, and this organisation establishes the basis for connectivity and function (Sanes and Zipursky, 2010). However, SEM analysis demonstrated that the IPL of *Lhx2-Cre:Tsc1^f/f^* mice was overgrown and densely packed. This phenotype bears gross morphological similarity to previous studies in which *Tsc1* ablation resulted in increased dendritic spine density in Purkinje cells and an increase in spine length and width in hippocampal pyramidal neurons (Tavazoie et al., 2005; Tsai et al., 2012). We therefore believe that conditional deletion of *Tsc1* in the eye leads to an increase in dendritic elaboration during development and that this aberrant neurite outgrowth is responsible for the densely packed nature of the IPL seen in *Lhx2-Cre:Tsc1^f/f^* mice. That rod bipolar cells were also observed to possess enlarged axon pedicles supports our hypothesis. Moreover, this overgrowth could also function as a physical barrier and contribute to the ectopic radial-migration phenotype discussed above. Furthermore, the stratification deficits seen for the amacrine cell subtypes might develop as a direct consequence of this dendritic overgrowth because sublaminae organisation could be compromised in *Lhx2-Cre:Tsc1^f/f^* mice. But what is also intriguing is that the aberrant IPL neurite targeting seen in our study is also reminiscent of reports in which transmembrane semaphorin or plexin signalling is perturbed during retinal stratification (Matsuoka et al., 2011a,b; Sun et al., 2013). Semaphorins mediate their effects through mTORC1 signalling (Campbell and Holt, 2001) and therefore changes to semaphorin-plexin signalling as a direct result of *Tsc1* ablation could also be a contributing mechanism for the sublaminae deficits reported here.

We observed accelerated RGC differentiation that is reminiscent to a previous *Drosophila* study (Bateman and McNeill, 2004). The authors demonstrated a loss of temporal neurogenic control upon *Tsc1* ablation that induced accelerated photoreceptor differentiation. Most importantly, no changes to cell specification were observed. The authors therefore concluded that loss of *Tsc1* does not alter cell fates but has an impact upon the temporal control of when cell fate decisions are made. It is therefore plausible that the accelerated differentiation of RGCs observed in our study arises from a similar mechanism. That we observed the expanded expression domains for several genes involved in RGC development (*Math5*, *Dlx1* and *Dlx2*) adds support to this hypothesis. Further studies into the mechanistic regulation of accelerated RGC neurogenesis in *Lhx2-Cre:Tsc1^f/f^* mice are required. But the recent demonstration that *Unkempt* and *Headcase* act in the same pathway to negatively regulate the temporal control of *Drosophila* photoreceptor differentiation downstream of *Tsc1*-mTOR provides an intriguing avenue for future investigation, particularly because both the unkempt and unkempt-like genes are expressed in the mouse retina (Avet-Rochex et al., 2014; Blackshaw et al., 2004).

RGCs project to the dLGN in a stereotypical manner, with retinogeniculate topography being established by a combination of EphA/ephrin-A signalling, patterned retinal-activity-dependent axonal refinement and competition (Feldheim et al., 1998; Penn et al., 1998; Torborg and Feller, 2005). Notable differences were observed in retinogeniculate topography of the *Lhx2-Cre:Tsc1^f/f^* mice. Firstly, an expected reduction in the total (contra- and ipsilateral) and ipsilateral-only territories was evident. This was presumably due to RGC apoptosis and the consequent disruption to mosaic regularity we observed in *Lhx2-Cre:Tsc1^f/f^* mice. Secondly, the topography and position of the ipsilateral patch within the dLGN of *Lhx2-Cre:Tsc1^f/f^* mice was altered and in some instances we observed more than one ipsilateral patch. This phenotype is reminiscent to the dLGN ipsilateral projection phenotype reported for nicotinic acetylcholine receptor β2 (*nAChRβ2*), *ephrin-A2/A3/A5* and *Phr1* mouse models (Culican et al., 2009; Pfeiffenberger et al., 2005; Rossi et al., 2001). Three possible mechanisms could account for this observed ipsilateral phenotype: (i) firstly, *Lhx2-Cre:Tsc1^f/f^* mice could have defects in axonal refinement. Previous studies have demonstrated that nAChRβ2-driven retinal waves during the first postnatal week of development are crucial for dLGN axonal refinement (Cang et al., 2005; Grubb et al., 2003; Rossi et al., 2001). *nAChRβ2*-null mice have defects in axonal refinement that are reminiscent to those observed in our study and therefore we cannot rule out the possibility that *Lhx2-Cre:Tsc1^f/f^* mice display a similar loss of retinal activity within the first postnatal week that contributes to the topographic phenotypes reported here. (ii) A second possible mechanism is that *Lhx2-Cre:Tsc1^f/f^* mice could have defects in EphA/ephrin-A-dependent axon-guidance mechanisms similar to that recently documented for *Tsc2* heterozygous mice. The authors demonstrated that RGCs in *Tsc2^+/−^* mice had aberrant ipsilateral projections and provided mechanistic data suggesting that TSC-mTORC1 signalling cooperates with the ephrin–Eph-receptor system to control projection topography within the dLGN (Nie et al., 2010). Our observations expand on this previous report and clearly demonstrate that the aberrant ipsilateral topography occurs through an RGC-autonomous mechanism because our fate-mapping experiments verified that the *Lhx2-Cre* transgene was not expressed in the dLGN (Fig. S7F). (iii) A third potential mechanism for the dLGN topographic phenotype observed in *Lhx2-Cre:Tsc1^f/f^* mice is one that is independent of retinal activity or EphA/ephrin-A signalling. A recent study reported that conditional deletion of a PHR protein family member (*Phr1*) led to eye-specific domains within the dLGN being severely disturbed in a manner similar to that observed in *Lhx2-Cre:Tsc1^f/f^* mice (Culican et al., 2009). The authors did not propose a specific mechanism for the observed retinotopic changes upon *Phr1* deletion, but this third mechanism is intriguing because PHR proteins are enriched in the CNS and have been demonstrated to interact and regulate activity of the hamartin-tuberin complex (Murthy et al., 2004).

In conclusion, we have generated a novel eye-specific model of TSC that recapitulates many of the neuropathological hallmarks of this syndrome: an increase in organ and cell size, ring heterotopias, hamartomas with retinal detachment, and lamination defects. Moreover, our results demonstrate a pivotal role for *Tsc1* in regulating various aspects of visual-pathway development and demonstrate that the TSC-mTORC1 pathway is critically involved in neuronal differentiation, migration and circuit formation. Our mouse model will provide a valuable resource for future studies concerning the molecular mechanisms underlying TSC and also as a platform to evaluate new therapeutic approaches for the treatment of this multi-organ disorder.

## MATERIALS AND METHODS

### Animals

All animal experiments were approved by the Animal Review Board at the Court of Appeal of Northern Norrland in Umeå. The generation and genotyping of *Tg(Lhx2-Cre)1Lcar* transgenic mice (referred to as *Lhx2-Cre*), *Tsc1^tm1Djk^* mice (referred to as *Tsc1^+/f^* or *Tsc1^f/f^*) and *Gt(ROSA)26Sor^tm1sor^* reporter mice (referred to as *ROSA26R*) have been described previously (Hägglund et al., 2011; Soriano, 1999; Uhlmann et al., 2002). The genotype of all animals was determined by PCR analysis of genomic DNA extracted from tail biopsies. Primers used to identify *Lhx2-Cre* and *ROSA26R* mice have been described previously (Hägglund et al., 2011). Primers used to identify *Tsc1^tm1Djk^* mice were IMR4008 5′-GTCACGACCGTAGGAGAAGC-3′ and IMR4009 5′-GAATCAACCCCACAGAGCAT-3′. Breeding *Lhx2-Cre:Tsc1^+/f^* and *Tsc1^f/f^* mice generated experimental animals. Breeding *Lhx2-Cre* and *ROSA26R* mice generated fate-mapping animals. The morning of the vaginal plug was considered as E0.5. Littermates lacking the *Lhx2-Cre* transgene that were either heterozygous or homozygous for the *Tsc1^tm1Djk^* allele were used as controls in all experiments. *Lhx2-Cre:Tsc1^f/f^* mice were born in Mendelian ratios (19 of 74 pups were *Lhx2-Cre:Tsc1^f/f^* in one set of genotyping experiments) and no gross eye defects were evident at birth. *Lhx2-Cre:Tsc1^f/f^* mice were indistinguishable from controls until P5. Thereafter, they failed to thrive and no *Lhx2-Cre:Tsc1^f/f^* mice survived longer than P21 (Fig. S9). The observed mortality presumably occurs owing to the activity of the *Lhx2-Cre* transgene and consequent ablation of the *Tsc1^tm1Djk^* allele in other domains outside the retina (Hägglund et al., 2011).

### Immunoblotting

The cornea, lens, ON and blood vessels were removed from enucleated eyes and the retina/RPE was snap-frozen in liquid nitrogen. Tissues were homogenized in SDS lysis buffer [100 mM Tris, pH 6.8; 2% (w/v) SDS], containing protease and phosphatase inhibitor cocktails (Complete Mini & PhosSTOP, Roche), using a TissueLyser (Qiagen Inc.). The protein extracts were centrifuged at 14,000 ***g*** for 5 min at 4°C and the concentration of the soluble fraction was measured using the BCA Protein Assay Kit (Fisher Thermo Scientific). Western blotting was performed on soluble protein extracts (10 µg) using Criterion TGX gels (Bio-Rad) and nitrocellulose membranes (Bio-Rad) as previously described (Mahmood and Yang, 2012). Reactive proteins were visualised using SuperSignal West Dura Extended Duration Substrate (Fisher Thermo Scientific). Imaging and quantification was performed using a ChemiDoc MP Imaging System (Bio-Rad). The following antibodies and dilutions were used: Hamartin (Cell Signaling Technology, 1:1000), S6 (Cell Signaling Technology, 1:1000), pS6 (S235/236) (Cell Signaling Technology, 1:1000), pS6 (S240/244) (Cell Signaling Technology, 1:1000) and GAPDH (Cell Signaling Technology, 1:30,000).

### Optical projection tomography (OPT)

Enucleated eyes were pierced through the cornea with a 27-gauge needle to allow for fixative infusion and prepared for OPT scanning as described previously (Alanentalo et al., 2007). Specimens were scanned in transmission OPT mode on a Bioptonics 3001 OPT scanner (Bioptonics) and the acquired data was processed as described previously (Cheddad et al., 2012). 3D volumes based on the tomographic reconstructions were rendered in Drishti software (Version 2.2).

### Histology

Heads or enucleated eyes were fixed in 2% (w/v) glutaraldehyde in PBS overnight at 4°C. Eyes were immersed in 70% (v/v) ethanol and paraffin-embedded. Paraffin sections (10 µm) were cleared by 2×5 min incubation in xylenes before rehydration through a graded series of ethanol [99.5%, 95%, 90% and 80% (v/v) in PBS]. Haematoxylin and eosin staining was performed as previously described (Hägglund et al., 2011).

### Immunohistochemistry

For cryosection analysis, heads or enucleated eyes were fixed in 4% (w/v) PFA in PBS for up to 2 h on ice, equilibrated overnight at 4°C in 30% (w/v) sucrose in PBS and embedded in OCT compound (Sakura Finetek). For flat-mount analysis, retinae were dissected out from enucleated eyes and individually placed in separate wells of a 24-well plate and fixed in 4% (w/v) PFA in PBS for 2 h on ice. Immunohistochemistry was performed on cryosections (10-20 µm) or flat-mount retinae as previously described (Cantrup et al., 2012; Ma et al., 2007). The following antibodies and dilutions were used: pS6 (S235/236) (Cell Signaling Technology, 1:100), Brn3 (Santa Cruz Biotechnology, 1:50), activated caspase3 (Abcam, 1:1000), NF (SMI31) (Covance, 1:1000), GFAP (Chemicon, 1:400), Pax6 (DSHB, 1:100), ChAT (Millipore, 1:100), Calretinin (Swant, 1:1000), PKCα (Santa Cruz Biotechnology, 1:100), Calbindin-D (Sigma-Aldrich, 1:100) and p27^Kip1^ (BD Biosciences, 1:750).

### Proliferation analyses

Pregnant mice were injected intraperitoneally with 5-bromo-2′-deoxyuridine (BrdU) at 50 µg/g body weight for 5 min before sacrifice and embryo collection. Embryonic heads were fixed in 4% (w/v) PFA in PBS for up to 2 h on ice, equilibrated overnight at 4°C in 30% (w/v) sucrose in PBS and embedded in OCT compound (Sakura Finetek). Cryosections (10 µm) were washed in PBS and then denatured in 2 M HCl for 30 min at 37°C. The slides were subsequently neutralised with 0.1 M borate buffer (pH 8.5) for 10 min at room temperature (RT) followed by immunohistochemistry as described above using an anti-BrdU antibody (BD Pharmingen, 1:20).

### *In situ* hybridisation

Heads or enucleated eyes were fixed in 4% (w/v) PFA in PBS for up to 2 h on ice, equilibrated in 30% (w/v) sucrose in PBS overnight at 4°C and embedded in OCT compound (Sakura Finetek). *In situ* hybridization on cryosections (10 µm) was performed as previously described (Schaeren-Wiemers and Gerfin-Moser, 1993). The following IMAGE clones (Source Bioscience) were purchased to generate *in situ* probes: *Crx* (Clone #4527863), *Dlx1* (Clone #30360273), *Dlx2* (Clone #5718422), *Math5* (Clone #6824509), *Tbx5* (Clone #30548234) and *Vax2* (Clone #40101825).

### Intra ocular pressure (IOP) measurement

IOP measurements were performed using a Tonolab instrument (Icare Finland Oy) according to the manufacturer's instructions.

### *Lhx2-Cre:ROSA26R* fate-mapping

Embryonic heads or enucleated eyes were harvested from *Lhx2-Cre:ROSA26* mice and fixed in 4% (w/v) PFA in PBS for up to 2 h on ice. The tissues were subsequently equilibrated in 30% (w/v) sucrose in PBS overnight at 4°C, embedded in OCT compound (Sakura Finetek) and coronal cryosections (10 µm) were prepared. Adult *Lhx2-Cre:ROSA26* mice (>6 weeks old) were transcardially perfused with 30 ml of ice-cold 4% (w/v) PFA in PBS. The brain was subsequently removed and embedded in 4% (w/v) agarose in PBS. Coronal vibratome sections (100 µm) were prepared. β-galactosidase staining was performed as described previously (Hägglund et al., 2011).

### Retinogeniculate projection analysis

Heads were fixed in 4% (w/v) PFA in PBS for 24 h at 4°C. The cornea and lens were removed from each eye and a 1-mm^2^ piece of NeuroVue Red Plus or NeuroVue Maroon (Polysciences Inc.) was inserted into the optic disc (Fig. S7A). The heads were incubated in 4% (w/v) PFA in PBS for 8 weeks at 37°C. The brain was subsequently removed and embedded in 4% (w/v) agarose in PBS. Coronal vibratome sections (100 µm) were counterstained with DAPI and mounted with Aqua Polymount (Polysciences Inc.). Retinogeniculate analyses were performed as described previously (Leamey et al., 2007) and were calculated across the entire rostrocaudal extent of the dLGN. The total area (µm^2^) of the dLGN (as determined by DAPI staining) was first calculated using the freehand selection tool in Fiji (Schindelin et al., 2012). Background was then subtracted using the rolling ball function and the area occupied by both the contralateral and ipsilateral retinogeniculate projections was calculated using the same approach as above for the total area analysis. The territory occupied by the ipsilateral input was subsequently calculated as a percentage of the total dLGN area.

### Scanning electron microscopy (SEM)

Enucleated eyes were pierced through the cornea with a 27-gauge needle to allow for fixative infusion and fixed in 2% (w/v) glutaraldehyde in 0.1 M cacodylate buffer (pH 7.2) overnight at 4°C. Eyes were subsequently immersed in 70% (v/v) ethanol and paraffin-embedded. Paraffin sections (10 µm) were cleared by 2×5 min incubation in xylenes. Slides were subsequently washed 2×5 min in 99.5% (v/v) ethanol and allowed to air dry. Dehydrated samples were attached onto aluminium mounts using carbon adhesive tape and coated with 5 nm gold/palladium. Section morphology was examined using a Zeiss Merlin field emission scanning electron microscope using a secondary electron detector at a beam accelerating voltage of 4 kV and probe current of 150 pA.

### Image analyses

Immunohistochemistry and NeuroVue images were captured using a Zeiss LSM 710 confocal microscope. Histology, *in situ* hybridisation and fate-mapping images were captured using a Nikon Eclipse E800 microscope fitted with a Nikon DS-Ri1 digital colour camera. All images were compiled and analysed using Fiji (Schindelin et al., 2012), CellProfiler (Carpenter et al., 2006; Kamentsky et al., 2011), Adobe Photoshop and/or Adobe Illustrator.

### Statistical analyses

All statistical analyses were performed using Prism6 (GraphPad Software). Unpaired two-tailed Student's *t*-tests were used to determine statistical significances. All statistical analyses were performed on data derived from at least three mice of each genotype. Error bars in all figures represent the standard error of the mean (s.e.m.). *P*-values are indicated as follows: ns, not significant; *P≤0.05, **P≤0.01, ***P≤0.001, ****P≤0.0001.
